# Assessing the reactions of tourist markets to reinstated travel restrictions in the destination during the post-COVID-19 phase

**DOI:** 10.1038/s41598-024-66459-2

**Published:** 2024-07-05

**Authors:** Xuankai Ma, Rongxi Ma, Zijing Ma, Jingzhe Wang, Zhaoping Yang, Cuirong Wang, Fang Han

**Affiliations:** 1 Urumqi Urban Institute of Geotechnical Investigation Surveying and Mapping, Urumqi, 830000 China; 2 State Key Laboratory of Desert and Oasis Ecology, Xinjiang Institute of Ecology and Geography, Chinese Academy of Sciences, Urumqi, 830011 China; 3 Institute of Geographic Sciences and Natural Resources Research, Chinese Academy of Sciences, Beijing, No. 818, Beijing South Road, Urumqi, 100101 Xinjiang China; 4 University of Chinese Academy of Sciences, Beijing, 100049 China; 5 College of Geography and Remote Sensing Sciences, Xinjiang University, Urumqi, 800046 China; 6 School of Artificial Intelligence, Shenzhen Polytechnic University, Shenzhen, 518055 China

**Keywords:** Tourism demand, Post-COVID-19, Tourism crisis management, Search engine data analysis, Tourism resilience, Socioeconomic scenarios, Sustainability

## Abstract

This study, leveraging search engine data, investigates the dynamics of China's domestic tourism markets in response to the August 2022 epidemic outbreak in Xinjiang. It focuses on understanding the reaction mechanisms of tourist-origin markets during destination crises in the post-pandemic phase. Notably, the research identifies a continuous rise in the potential tourism demand from tourist origin cities, despite the challenges posed by the epidemic. Further analysis uncovers a regional disparity in the growth of tourism demand, primarily influenced by the economic stratification of origin markets. Additionally, the study examines key tourism attractions such as Duku Road, highlighting its resilient competitive system, which consists of distinctive tourism experiences, economically robust tourist origins, diverse tourist markets, and spatial pattern stability driven by economic factors in source cities, illustrating an adaptive response to external challenges such as crises. The findings provide new insights into the dynamics of tourism demand, offering a foundation for developing strategies to bolster destination resilience and competitiveness in times of health crises.

## Introduction

The COVID-19 pandemic has had an enormous and long-lasting social and economic impact on the tourism industry^1^. International tourist arrivals (overnight visitors) plummeted by 73% in 2020 due to a global embargo, widespread travel restrictions, and a massive drop in demand. Compared to 2019, there were approximately 1 billion fewer international visitors that year^2^. The year 2021 is considered by scholars as the Year of Recovery for tourism, with tourism indicators signaling recovery^3,4^. The climate crisis and the European return to war are expected to restrict international travel^5^, and the domestic market will be an appropriate approach to drive tourism recovery^6^. The expansion of domestic tourism is propelled by a considerable accumulation of suppressed demand, a rising preference for domestic locales, and stringent border entry and exit controls.

Nevertheless, the mutation of the Omicron virus resulting in greater contagiousness will lead to localized areas being re-closed to counter the diffusion of the Virus^7^. It is an additional shock to the recovering tourist destinations. The investigation of the response of origin markets to the reclosure of destinations during the tourism recovery period is of significant relevance for regional authorities to assess the impact of the outbreak and the maintenance of tourist origins.

This paper aims to investigate what changes occur in origin market demand for destinations affected by reclosure during the tourism recovery period, what spatial differences such changes show, and how the differences in response exhibited by origin markets arise. The response in tourism demand is quantified through the analysis of annual growth rates in the domestic market during periods of reclosure. Based on tourism demand theory, demographic, economic, and environmental conditions, COVID-19 epidemic status, healthcare conditions, Internet access coverage, and traffic activation will be potential influential factors to account for the variation in origins market response. We add new insights to the COVID-19 study of short-term effects and spatial differences in tourism demand by examining the pattern of origins market response in the face of destination reclosure from the tourist origins' perspective (cities).

## Literature review

### The COVID-19 and the tourism industry

The dramatic impact of COVID-19 on the tourism industry has intensified the involvement of scholars in studies on the issue. In the initial phase of the pandemic (2020), UNWTO reported international tourist arrivals to decline by 70–75% for 2020. Academics have addressed the effects of the pandemic on national tourism markets^8,9^, the response of the tourism industry to the crisis^10,11^, and the framework for sustainable development^12–14^. Greece, with its developed tourism industry, the loss of tourism revenue due to the pandemic has directly contributed to the overall economic decline of the country^15^. The pandemic has declined tourist arrivals and affected employment and productivity in the Balearic Islands, revealing the region’s decline^1^. Hotel revenues in Italy fell by 66% relative to Turkey due to the embargo policies^16^. The U.S. hotel industry lost about $30 billion in revenue in the spring of 2020^17^, while the restaurant industry also faced a notable crackdown^18^. The multidimensional assessments of the influence of the pandemic on tourism demand were performed by comparing this crisis with the normal state of affairs before the crisis, which pointed to a significant negative consequence of tourism with its sibling sectors along the entire tourism industrial chain^19^. Restoring tourist arrivals to pre-crisis standards will probably confront prolonged pain^20^.

Some improvement in the epidemic accompanies the crisis response and recovery period (after 2021). Regardless, the pace of recovery continues to be sluggish and uneven in all regions of the world due to varying degrees of mobility restrictions, vaccination rates, and traveler confidence. Dynamic adjustment of travel restrictions within the country during the post-pandemic period could stimulate a recovery to a limited extent and allow domestic tourism to dominate^21,22^. Empirical studies show that regions with monoculture industries have no buffers to adapt to the crisis in the face of shocks that significantly reduce tourism demand^23,24^. In contrast, regions with prosperous industries and strong tourism specialization will be highly resilient to disruptions in the context of less restrictive movement of people, presenting a competitive advantage with a slight decline in demand^25,26^. Scholars believe this is the best time to promote equitable and sustainable tourism development, and the disconnect between tourist demand and destination development (growth expansion) needs to be repaired^27,28^. The construction of tourism sustainability will focus more on the changing needs of tourists themselves and their demand preferences. In the future, regulating the balanced development of regional tourism by changing tourists’ needs is the focus of tourism sustainability in the post-epidemic era^29,30^.

### Measures of tourism recovery

In the tourism literature, scholars have proposed and implemented the concept of tourism resilience to quantify post-crisis tourism outcomes, particularly the ability and magnitude of regional tourism to recover from the COVID-19 disruptions^31^. Metrics such as employment rates, tourist arrival rates, and tourism revenues are measures to assess regions’ tourism resilience within geographically large areas^32–34^. It is noteworthy that the recoverability of tourism in different regions varies by geographic area and thus shows distinct patterns of recovery. Thus, the spatial heterogeneity of tourism recovery must be considered^35^. Scholars have argued that location quotients^36^, the share of an industry in the local total divided by the share of the industry in the national total, can better account for the speed of tourism recovery from that region’s crisis^37^.

### Tourism demand change observation

In the era of Big Data, the utilization of search engines by tourists to acquire travel-related information ahead of their trips has become the initial step in travel planning^38^. The significance of search engine data in characterizing and predicting tourism demand has been gradually acknowledged by tourism researchers since 2010 and has been incorporated into various models for analytical work^39–42^. Due to the search behavior of tourists based on their destinations of interest, these electronic behavior records are normalized into search engine indexes to indicate tourism demand, preferences, travel intentions, and the location of the tourists’ origin^43^. Researchers have conducted studies on tourism demand response to pandemics and tourism recovery rates with search engine data, confirming the significant advantages of search engine data in fitting economic indicators during the COVID-19 pandemic^22,44,45^. Therefore, we attempted to characterize the rate of change in tourism demand in the origins market by investigating search engine data for a specific period (destination reclosure) as search engine data gives high-frequency information on the travel intentions of Internet users in different regions for a defined destination.

### Overview summary

In the post-epidemic era of tourism recovery, a sub-black swan event of local reclosure is inevitable. We are interested in how origin markets will respond to destination reclosure, the characteristics of the spatial response patterns, and the reasons for this heterogeneity that need to be urgently explored. While the literature has usefully explored the recovery of tourism with mature domestic markets, it is crucial to focus on the changes in tourism demand for destinations at the national level, where the origin markets are the roots of tourism demand. To address the limitations of previous research, this paper contributes to the tourism literature by using a search engine to quantify short-term tourism changes during tourism crises and constructing models of tourism demand changes in different regions to explore the drivers of different response patterns.

## Materials and methodologies

### Tourism demand data

Search engine data is identified as an efficacious data source for measuring high-frequency tourism demand. Google Trends has extensive applications worldwide, whereas Baidu has a more indicative role in China. The Baidu search engine has two data products: Keyword Search Index and Brand Index (https://index.baidu.com). The Keyword Search Index contains data from 2011 till now, and the Brand Index is a more professional industry-based index that will be available from August 2021. This paper utilizes daily keyword indices from 2011 to 2022 to investigate the background of tourism demand for the Duku Road and brand indexes from August 10 to October 31 in 2021 and 2022 to measure changes in tourism demand for the Duku Road from different origins regions during the destination closure period under the impact of the pandemic.

1 $${TD}_{s}^{t}= \sum BBI(i,j)$$

where *i* is a tourist origin city; *j* is a date from August 10 to October 31; *BBI* is the Baidu Brand Index of a city search for Duku Road on a date. *s* is a spatial series of origin cities or an individual city; *t* is a temporal series of dates; T.D. is the sum of demand from *s* during *t*.

### Study case

The Chinese domestic tourism market was chosen as the study case due to the robust intervention policies adopted by China during the first pandemic, dramatically impacting international and domestic tourism in China. In the post-epidemic era, a multitude of countries have eased international travel restrictions. The massive mobility of individuals within China and the mutation of the Virus made travel policies contingent on the consequences of the containment of the pandemic. In this context, domestic tourism demand has emerged a robust recovery. This phenomenon is also prevalent in other countries, and scholars are confident that domestic tourism will be the recovery engine in the short term. It is a matter of significant concern that tourist arrivals may abruptly decline to zero in instances where destinations are compelled to shut down anew amidst a pandemic resurgence. Notwithstanding, it is pertinent to note that latent tourism demand persists. In response, this study meticulously tracks variations in demand at these destinations and corresponding responses from origin markets, employing high-frequency search engine data as a quantitative metric of demand. Distinct from conventional statistical methods, the monitoring of such rapid changes necessitates the utilization of big data analytics.

Xinjiang was one of the most popular destinations for domestic tourism in 2022, with millions of tourists entering Xinjiang by self-drive, high-speed rail, and air. The Duku Road (high-rank landscape driveway) is the most attractive destination for tourists, with 28.35% of tourism demand in Xinjiang. It serves as a tourist hub, radiating tourists to other attractions^46^. Tourism prosperity has made Xinjiang an unignorable destination during domestic tourism recovery. Nevertheless, the massive tourist flows have planted the potential for the spread of the epidemic. The entire territory of Xinjiang entered a region-wide silent management to control the epidemic on August 10 and will remain in effect through the winter. During this period, all travel activities had to be ceased, and the government organized the transfer of healthy travelers back to their origins. This unexpected outbreak has had a fatal impact on Xinjiang’s thriving tourism industry. The study case has typical implications. This paper investigates the origins market’s response to the destination’s reclosure, considering the Duku Road as the destination and cities outside Xinjiang as the domestic origin markets.

**Figure 1:**
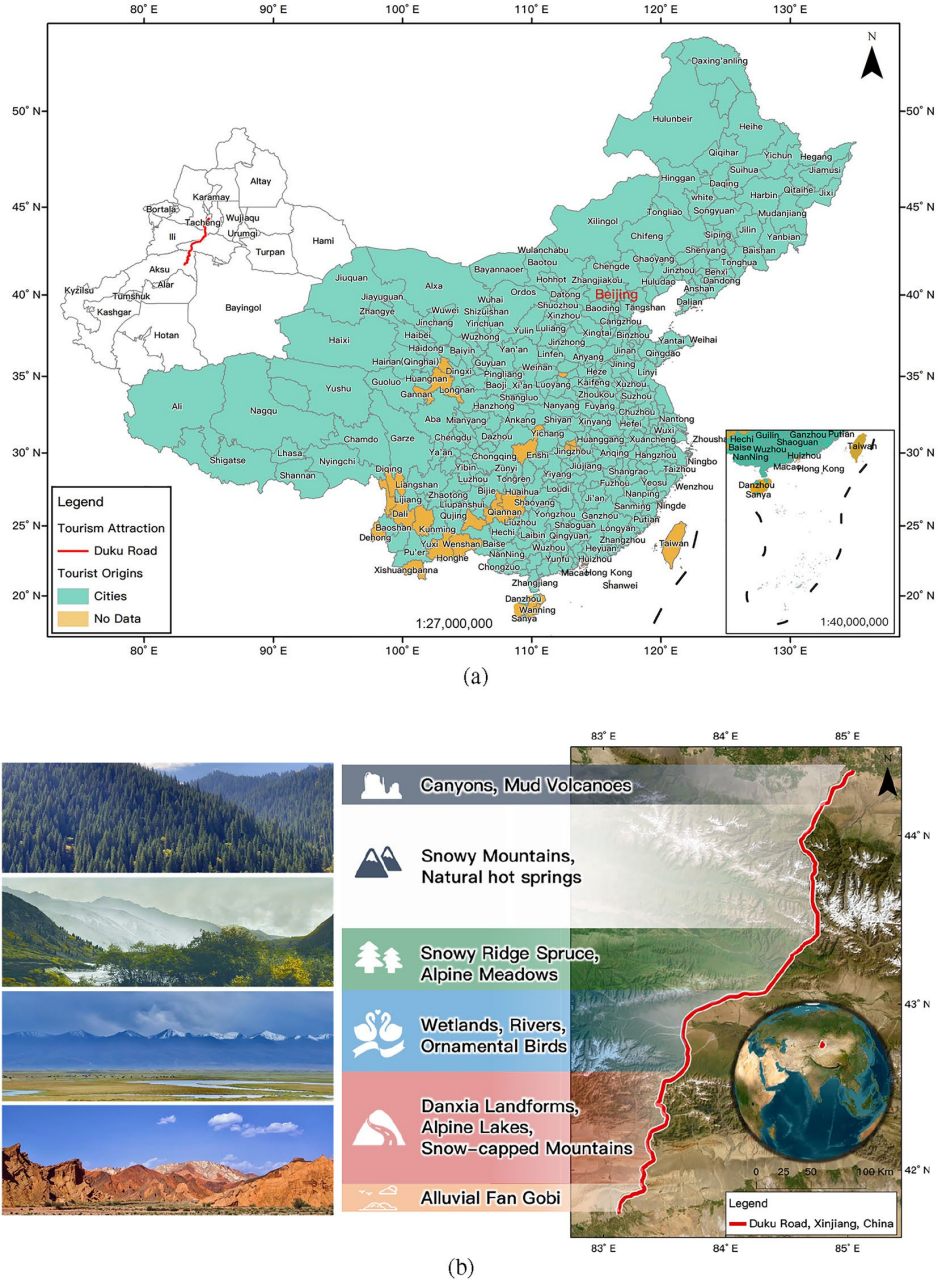
The study case. (**a**) The domestic tourist markets of Duku Road. (**b**) The location of Duku Road, Xinjiang, China. Notes: The map in (**a**) was produced using ESRI ArcMap 10.2, and the standard map service was provided by the Ministry of Natural Resources of China, accessible at http://bzdt.ch.mnr.gov.cn/ with Grant No. GS (2021) 5448. The photographs in (**b**) were taken by the first author for tourism resources investigation in September 2021.

Three hundred and seven cities of China’s domestic tourism market were adopted as the tourist origins for the case study. The Duku Road, located in Xinjiang, served as the destination (Fig. 1a). The Duku Road is a mysterious and fascinating landscaped driveway that stretches through the north and south of the Tianshan Mountains, a World Natural Heritage Site, which is also known as the Tianshan Road. The Duku Road runs with a unique topography, with numerous sharp curves and steep slopes, more than 280 km of road sections above 2000 m above sea level, 1/3 of the whole course is a cliff, 1/5 of the lot is in the high mountain permafrost, crossing nearly ten major rivers in the Tianshan Mountains, and over four ice-pass of mountains that accumulate snow all year round (Fig. 1b). Driving on Duku Road, travelers experience the seasonal transformations in a single day, which shows the magnificent scenery of “four seasons in one day, ten miles in different skies” to off-road enthusiasts and self-driving tourists.

### Dependent variables

**Figure 2:**
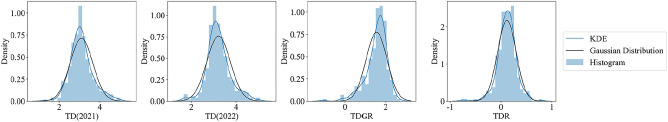
Statistical distribution analysis of TD, TDGR, and TDR variables. Notes: TD. is the Baidu brand index during the closure period, metric tourism demand, and the number in parentheses is the year; TDGR is the growth rates of tourism demand, and TDR is 2022 vs. 2021 ratio of tourism demand. The values of the above variables are processed by f = ln(x).

The literature has proposed numerous valid measures of change in tourism demand, and scholars have characterized tourism resilience based on micro-level expenditure elasticities and annual percentage changes between the pre-and post-pandemic periods^6,25^. This paper pays attention to measuring the instantaneous response of origin markets to the public health crisis in a destination that caused the closure period (August 10–October 31), where typically, growth rates are employed to capture the difference in performance relative to a benchmark^26,47^. Thus, tourism demand in 2021 is considered the initial period of the post-pandemic recovery period, and the growth rate for the corresponding period in 2022 versus the benchmark period is adopted as a measure of Tourism Demand Growth Rates (TDGR). The demand ratio is chosen indicator of short-term resilience^48^, and we generate the Tourism Demand Ratio (TDR) as the alternative dependent variable to test the robustness of conclusions. To illustrate the distribution of these dependent variables, Fig. 2 presents a histogram, kernel function density estimation plot, and maximum likelihood Gaussian distribution fit, providing a comprehensive view of the underlying data structure and the variability in Tourism Demand Growth Rates (TDGR) and Tourism Demand Ratio (TDR).

2 $${TDGR}_{i}=\frac{{TD}_{i}^{t{\prime}}-{TD}_{i}^{t}}{{TD}_{i}^{t}}\times 100\%$$

3 $${TDR}_{i}=\frac{{TD}_{i}^{t{\prime}}}{{TD}_{i}^{t}}$$

where *i* is a tourist origin city; *t'* is the current year (2022), and *t* is the control year (2021); TDGR*i* is the tourism demand variation of the city *i;* ${TD}_{i}^{t}$ is the sum of demand from city *i* in year *t*.

### Determinants and proxy variables

The willingness of tourists to explore a destination depends on the determinants of the origin’s financial capacity, physical constraints, and psychological tendencies, in addition to the destination’s attractiveness. Furthermore, challenged by pandemic restrictions on mobility, we considered the origin markets’ epidemic circumstances and medical availability. We have selected the population, economics, environmental conditions, COVID-19, medical healthcare, Internet accessibility, and traffic activeness of the origin cities as potential determinants of tourism demand to analyze their impact on tourism demand changes, as detailed in Table 1^43,49–53^.

**Table 1:** Determinants and their proxy variables.

Determinant	Proxy variable	Abbreviation	Unit	Year
Population	Population census	P1	RMB Yuan	2020
Economy	GDP per capita	E1	RMB Yuan	2020
	Disposable income per capita	E2	RMB Yuan	2021
	Consumption expenditure per capita	E3	10e4RMB Yuan	2020
	Average wage	E4	RMB Yuan	2020
	Consumer price index	E5	%	2020
	Savings deposits	E6	10e9 RMB Yuan	2020
Environmental conditions	PM2.5	EC1	μg/m^3^	2022
	AQI index	EC2	–	2022
	Industrial solid waste utilization rate	EC3	%	2019
	Living waste harmless disposal rate	EC4	%	2020
COVID-19	Confirmed	C1	–	2022.08–2022.10
	New confirmed	C2	–	2022.08–2022.10
	New asymptomatic	C3	–	2022.08–2022.10
	Dead	C4	–	2022.08–2022.10
	Healed	C5	–	2022.08–2022.10
Healthcare	Number of hospitals	H1	–	2020
	Number of hospital beds	H2	–	2020
	Number of basic medical insurance participants	H3	–	2020
Internet access	Number of mobile phone subscribers	IA1	10,000	2019
	Number of broadband access subscribers	IA2	10,000	2019
Traffic activation	Private vehicle	TA1	1000	2019
	Road passenger traffic	TA2	10,000	2019
	Air passenger traffic	TA3	10,000	2019
	Spatial distance	TA4	km	2021

**Figure 3:**
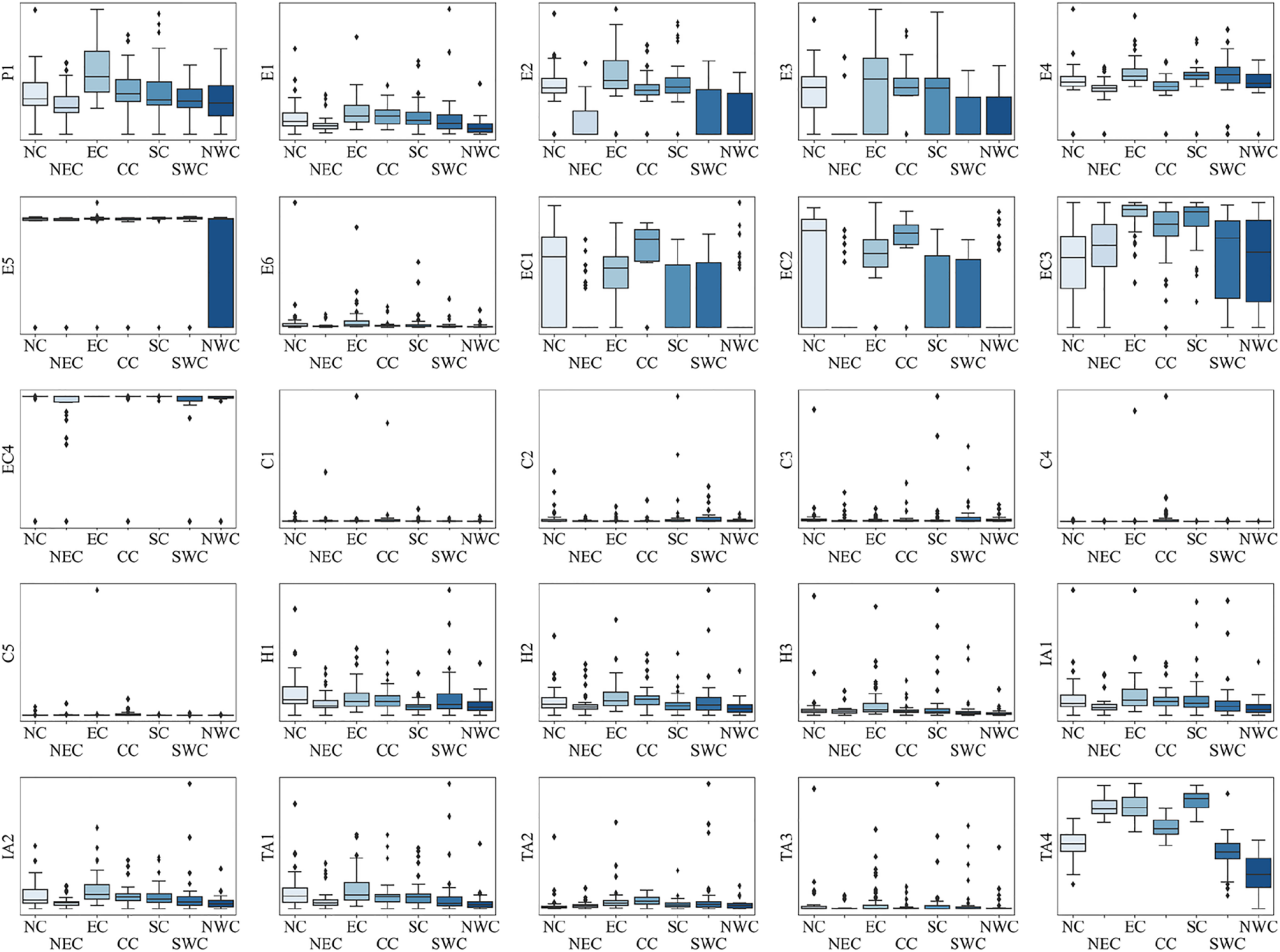
Proxy variable box plots group by regions. Notes: The labels of the X-axis, East China, South China, Southwest China, North China, Central China, Northeast China, and Northwest China are abbreviated as N.C., NEC, E.C., CC, SC, and NWC, respectively. The Y-axis labels are abbreviations for the proxy variables in Table 1.

China has a substantial tourism market with spatially non-stationary development levels of cities in each region, and origins cities have significant spatial stratification heterogeneity. Accordingly, following large administrative geographical regions, we disaggregate the origin markets into East China, South China, Southwest China, North China, Northeast China, and Northwest China. The statistical data of each city are obtained from the National Bureau of Statistics (http://www.stats.gov.cn/), and the epidemic data sources of COVID-19 are informed by the National Health and Health Commission and the provincial health and health commissions (https://2019ncov.chinacdc.cn/2019-nCoV/). The advantages of this paper’s dataset are that it measures the seven explanatory variables mentioned above through twenty-five proxy variables, which captures the diversity of factors driving the tourism demand changes. Additionally, all the proxy variables will be standardized within large administrative geographical regions (Fig. 3), which helps to reveal significant differences in the explanatory variables performing distinct roles in different local regions.

### Research framework

**Figure 4:**
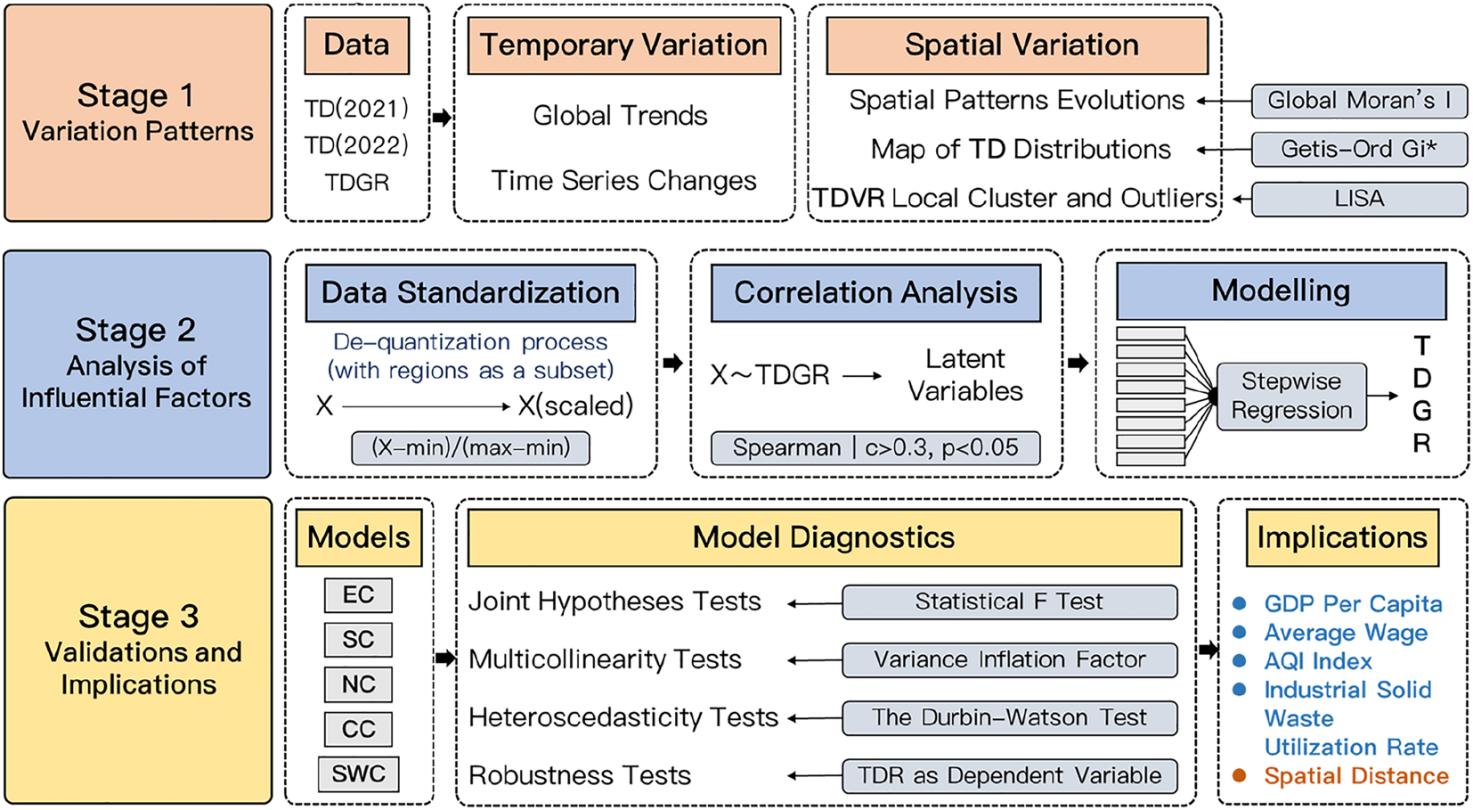
The research framework. Notes: TD-Tourism Demand; TDGR-Tourism Demand Growth Rates; LISA- Local Indicators of Spatial Association; EC, SC, NC, CC, SWC are the models in different regions (East China, South China, North China, Central China, and Southwest China) respectively.

The research framework, as illustrated in Fig. 4, consists of three stages: temporal and spatial change analysis of tourism demand, exploratory regression analysis, and model evaluation.

The first stage involves depicting the spatial pattern of the Duku Road tourist origin markets and its changing process in the temporal dimension. The Global Spatial Autocorrelation method is applied to assess the spatial patterns of the origin markets, the Getis-Ord Gi* Hotspot Analysis method^54^ detects the dominant origin market clusters, and the Local Spatial Autocorrelation Analysis method^55^ is assigned to reveal clusters and outliers areas of tourism demand variation. The spatial distribution characteristics will map out the origin markets and pinpoint the dominant origin regions.

In the second stage, this paper uses an exploratory knowledge discovery strategy, i.e., finding the most significant drivers of each region among the many proxy variables that may impact tourism demand changes. Candidate proxies that are significantly correlated are initially filtered out from the Correlation Analysis between the proxies and explanatory variables. Then the candidate proxies are imported into a multi-round Linear Regression Analysis for iterative modeling and comparisons, thereby investigating the factors influencing the tourism demand variation in each region. Since the proxy variables in this paper are not entirely customarily distributed, Spearman’s Correlation Analysis^56^ has the advantage of handling mixed data. The proxy variables with correlation coefficients greater than 0.3 and a significance over 95% will be considered candidate proxy variables. The Stepwise Regression Analysis^57^ can perform multiple turns of regression analysis on candidate proxy variables and automatically remove the insignificant variables, resulting in the exploration of the model with the optimal performance with significance.

The third stage is the diagnostic evaluation and robustness testing of the model. Multiple linear regression, autocorrelation, normality of residuals, and eteroscedasticity are used to diagnose the model’s performances^57^. We compare the conclusions obtained from Stepwise Regression Analysis with the results obtained by substituting the dependent variable to evaluate the validity of the conclusions. The above experiments will be replicated with the ratio of tourism demand during the destination closure in 2022 to the corresponding period in 2021 as the dependent variable, and the conclusions will be considered dependable and robust in case of substantial consistency.

## Results

### Spatial and temporal changes in origin markets demand

**Figure 5:**
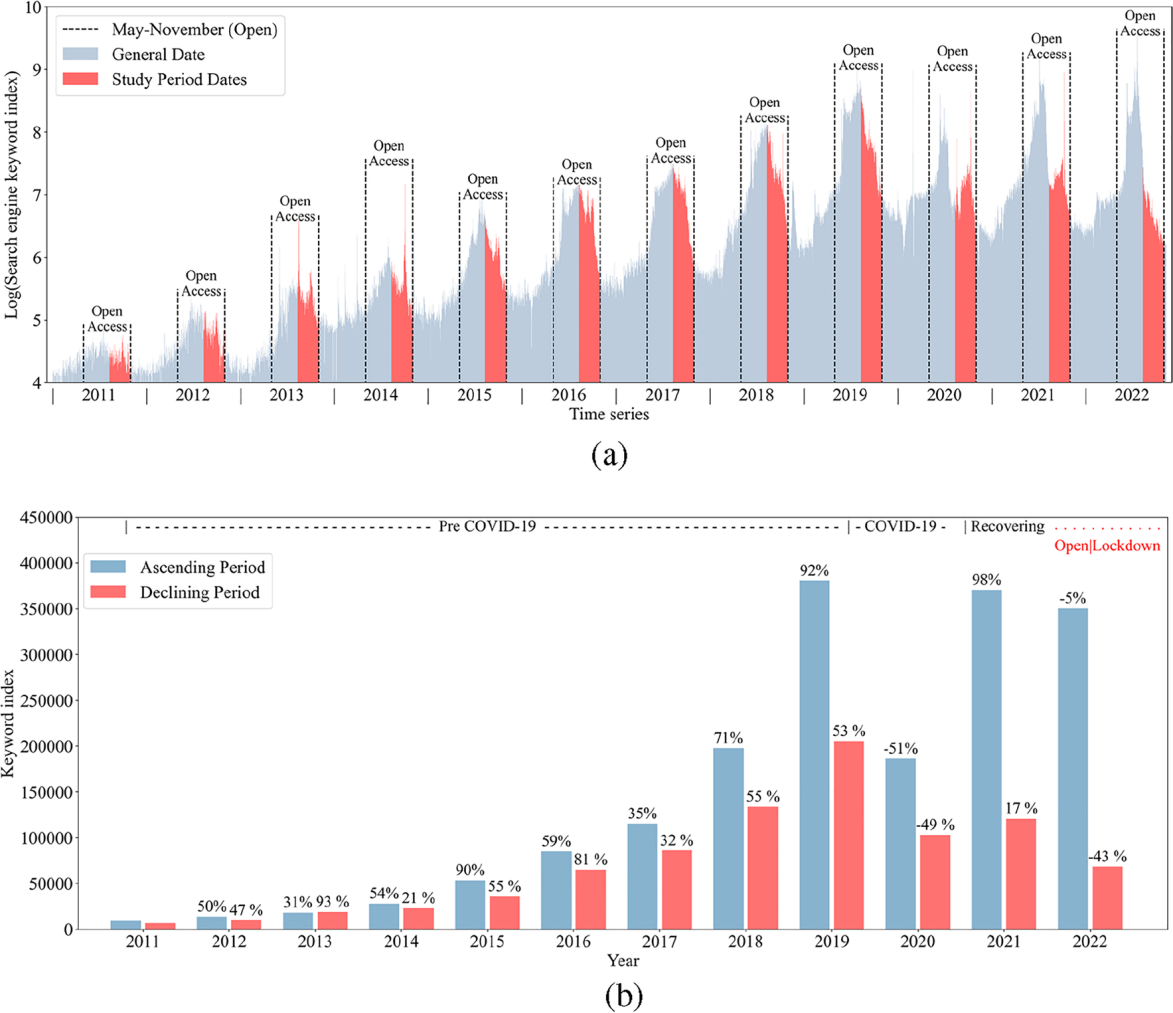
Variation of Baidu index for Duku Road (2011–2022). (**a**) The daily Baidu Index trend for Duku Road (2011–2022). (**b**) The Baidu Index during the years of unrestricted access for The Duku Road (2011–2022).

The paper utilizes the Baidu search engine to garner search records of the Duku Road in China over the past decade (Fig. 5a). Results indicate a rapid surge in the attraction of the Duku Road since 2015, peaking in 2019. Despite the impact of COVID-19 in 2020, the demand for tourism quickly rebounded, hitting a new high in 2022. It is imperative to note that due to safety risks associated with the Duku Road, it is only accessible to tourists from May to October every year. During this period, the tourism demand for Duku Road exhibits a primary and secondary peak, with a critical point at the main peak, dividing this phase into a rising and a declining period. Specifically, the rising period commences in early May, peaking towards the end of July, followed by a continual decline. However, a second surge forms during the National Day Golden Tourism Week, which subsequently dwindles gradually.

Considering a sudden outbreak of COVID-19 across Xinjiang, local authorities initiated a silent management policy, imposing traffic restrictions from August 10 to October 31. According to historical time series characteristics of the Duku Road, this time point coincides closely with the critical date when the tourism demand transitions from a peak to a decline. A comparison of the keyword index during the rising and declining periods of each year reveals that the tourism demand maintained a high level before the travel restrictions, while the destination closure significantly impacted the tourism demand, causing a sharp decline (Fig. 5b).

Although post August 10, 2022, marked the declining phase of tourism demand for the Duku Road, the Baidu index for the keyword “Duku Road” remained high (only a 5% decrease compared to the same period in 2021) before August 10, 2022. This suggests a stable tourism demand for the Duku Road prior to the pandemic closure. However, post August 10, the Baidu index plummeted by 43% compared to the same period in 2021, inferring a direct impact on the tourism demand for the Duku Road due to the outbreak of COVID-19 in Xinjiang and the traffic control measures. Noteworthy is that despite the inability to conduct any tourism activities in Xinjiang post August 10, the source market maintained a robust potential tourism demand for the Duku Road from August 10 to October 31. During the COVID-19 outbreak and lockdown in Xinjiang, the tourism demand from seven administrative regions in China for the Duku Road grew by an average of 31.49% compared to 2021, indicating a strong resilience in the tourism demand for the Duku Road. This reflects that despite the restrictions imposed by the pandemic and traffic control policies, the restrained tourism demand will gradually be released once the situation is under control, highlighting a significant potential for recovery.

Based on the spatial autocorrelation analysis results (Table 2), the tourism demand from the source market demonstrates a significant spatial clustering pattern, with the spatial pattern of the Duku Road’s source market transitioning from a low level to a moderate level of spatial clustering. The spatial distribution of tourism demand from source cities to the Duku Road has been impacted by the pandemic crisis, exhibiting a trend of contraction and clustering towards core source cities.

**Table 2:** The spatial pattern of origin markets.

	Global Moran's I summary			Diagnostic parameters	
	TD (2021)	TD (2022)	TDGR (2022 vs. 2021)	Significance level	Critical value
Moran's Index	0.038	0.047	0.080	0.01	[− ∞, 2.58)
Expected Index	− 0.003	− 0.003	− 0.003	0.05	[− 2.58, − 1.96)
Variance	0.001	0.001	0.001	0.1	[− 1.96, − 1.65)
z-score	1.695	2.105	3.214	0.1	(1.65, 1.96]
p-value	0.090	0.035	0.001	0.05	(1.96, 2.58]
Spatial pattern	Lowly clustered	Moderately clustered	Highly clustered	0.01	(2.58, + ∞)

*TD* tourism demand during the 2022 reclosure and the same period in 2021, *TDGR* tourism demand growth rates.

**Figure 6:**
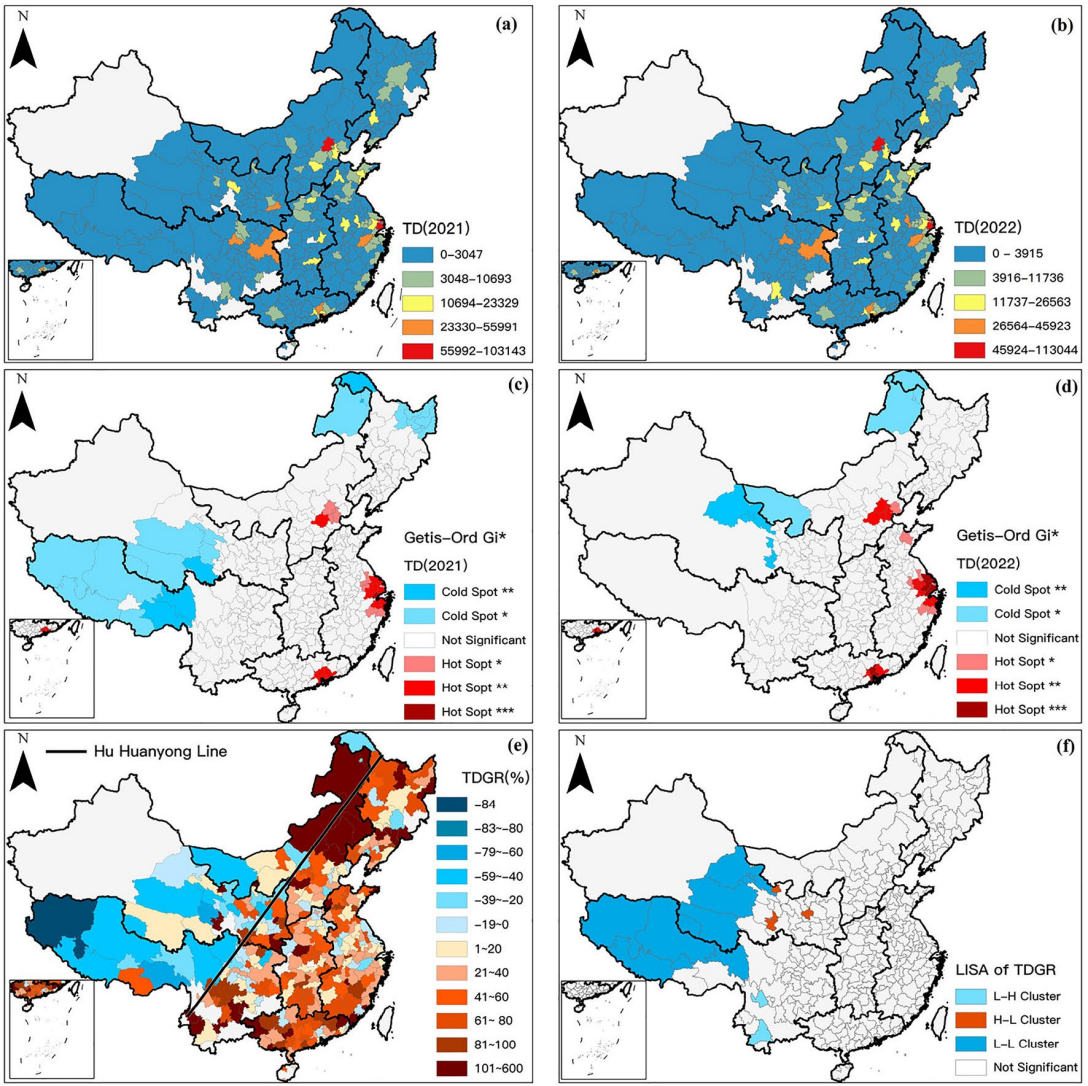
The spatial patterns of tourism demand and its' variation. Notes: Hu Huanyong Line in (**e**) is a comparison divider proposed by Chinese geographer Hu Huanyong (1901–1998) in 1935 to delineate the population density of China, which has significant heterogeneity in the population, society, and economy between the east and west of the line.

Viewed from the perspective of source cities, this section utilizes the Jenks Natural Breaks method to categorize source cities into five groups based on the intensity of tourism demand towards Duku Road and constructs a spatial distribution map of tourism demand from the source market. The map employs a color gradient from blue to red to represent the intensity of tourism demand from source cities to the Duku Road, where red and orange cities signify core and secondary source cities, respectively. The domestic source market is primarily distributed in the developed cities in eastern China. In Fig. 6a,b, cities like Beijing and Shanghai are identified as core source cities, while Chengdu, Chongqing, Hangzhou, and Guangzhou are recognized as secondary source cities. A comparison between Fig. 6a,b reveals that cities like Lanzhou, Xi’an, and Nanjing no longer serve as secondary source locations, indicating that some secondary source cities are more sensitive to the tourism demand for Duku Road under Xinjiang pandemic crisis than others, which exhibiting a variability within the secondary source market.

Upon further hotspot analysis of the source market, the results exhibit significant statistical clustering in the spatial domain for the Duku Road’s source market. As depicted in Fig. 6c,d, the red zones represent the hotspots of tourism demand, encompassing the Jing-Jin-Ji region, Yangtze River Delta, and the Pearl River Delta, which emerge as the most crucial source markets with lesser impact from the pandemic crisis on Duku Road. On the other side, the blue zones signify the cold spots of tourism demand, where cities within these regions exhibit an exceedingly low tourism demand towards the Duku Road. A comparative inspection of Fig. 6c,d unveils a shift in some cold spot areas (for instance, the disappearance of the cold spot on the Qinghai-Tibet Plateau, and the emergence of a new cold spot area north of the Hexi Corridor), while the stability of cold spot regions in Northeast China remains high (such as Hulunbuir and Daxing’anling area).

This section analyzes the growth rate of tourism demand, designating cities with growth rates below zero with a gradient of cool colors (blue), and those with growth rates above zero with a gradient of warm colors (red), with intervals of (±) 20%. As shown in Fig. 6e, cities with a growth rate exceeding 100% are rendered in deep red. There is a significant positive correlation in the spatial distribution of tourism demand growth rates, indicating neighboring cities share a consistent response pattern to the pandemic. A distinct contrast is formed on either side of the Hu Huanyong Line; source markets to the east primarily exhibit positive growth rates, while those in the western regions display negative growth rates. Analysis of local clustering and outliers reveals the formation of three distinct local clusters in the spatial distribution of tourism demand growth rates (Fig. 6f). Cities near the Qinghai-Tibet Plateau and Hexi Corridor in Northwestern China respond strongly to the pandemic crisis, with rapid declines in tourism demand towards Duku Road in this area (L–L). A sporadic distribution of outlier cities (H–L) emerges on the eastern side of Northwestern China, exhibiting superior risk resilience and higher tourism demand compared to other local areas. In Southwest China, Yunnan province has higher demand growth, with Pu’er and Lijiang being typical tourist cities. The fluctuations in their demand are impacted by their respective tourism industries, exhibiting a phenomenon where they are encircled by proximate cities experiencing high demand growth rates (L–H).

**Figure 7:**
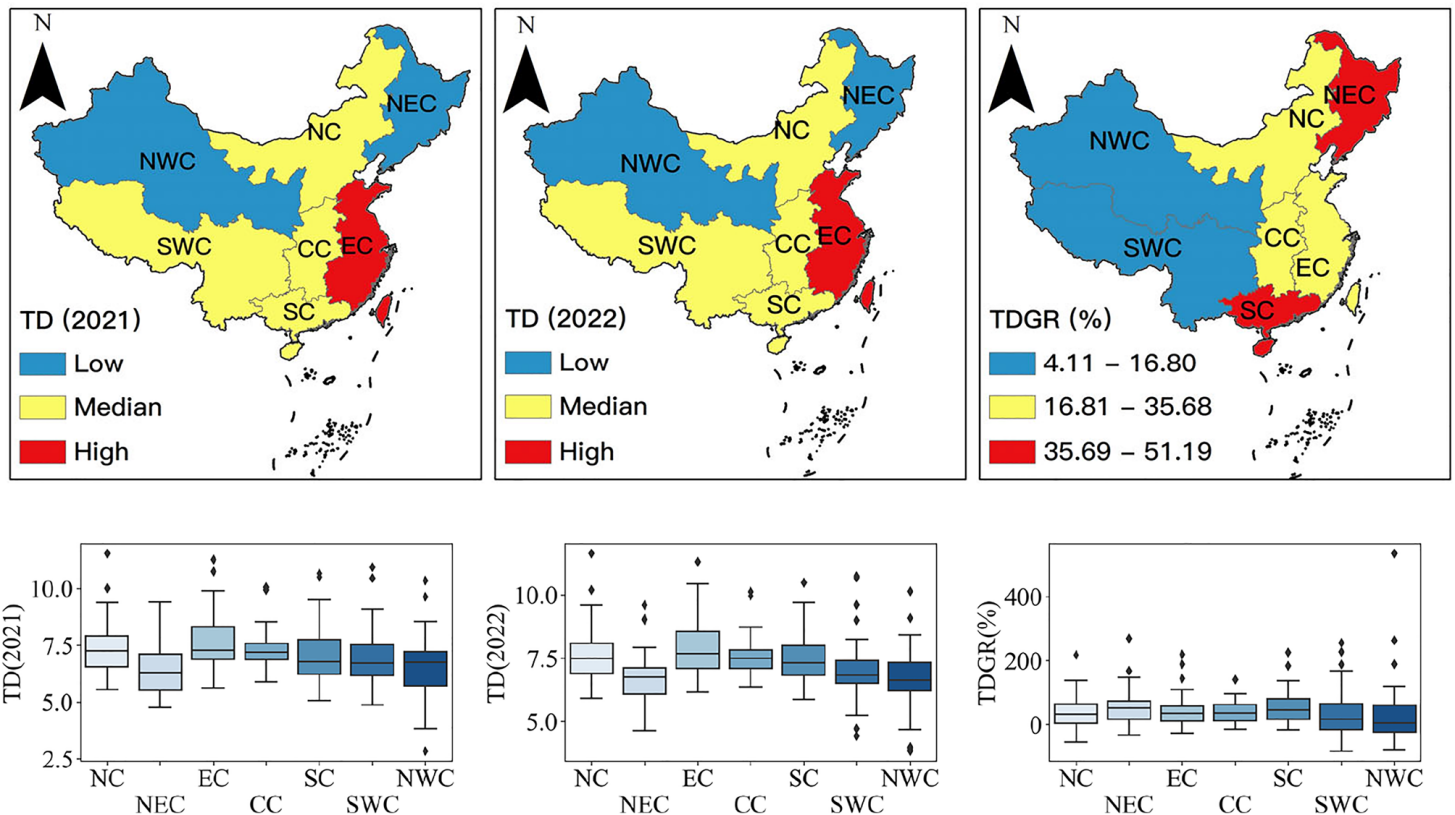
The maps with box plots of the tourism demand and growth rates.

By dividing the source market according to administrative regions, summing up the tourism demand from cities within each administrative region to Duku Road, and averaging the growth rate of tourism demand, regional differences are identified. As shown in Fig. 7, a comparison of tourism demand scale and growth between 2021 and 2022 across administrative regions is made. Using the Jenks Natural Breaks method, they are categorized into high, medium, and low levels. This corresponds to East China being the primary source market for Duku Road, while North, Central, South, Southwest China are secondary source markets, and Northeast and Northwest China are potential source markets. Northeast and South China have the highest growth rates in tourism demand, followed by North, Central, and East China, with Southwest and Northwest China having the lowest. There is minimal variation in the scale of tourism demand from cities within each region to Duku Road, but a larger difference in the growth rates of tourism demand.

### Correlation analysis for TDGR

The correlation between explanatory variables and the growth rate of tourism demand significantly varies across different regions, suggesting that a more nuanced analysis could be obtained by partitioning the source market for modeling. In the South China region, 64% of the explanatory variables are negatively correlated with the growth rate of tourism demand, while in the Northwest region, no explanatory variables exhibit significant correlation. In the Northeast region, only two explanatory variables are negatively correlated with the tourism demand growth rate. In East China, Southwest, North China, and Central China, about one-third of the explanatory variables show a correlation with tourism demand growth rate at a significance level better than 0.05.

The correlation results indicate that under the “dynamic zeroing” policy control, new cases in source areas do not correlate with the potential tourism demand growth rate of residents. For instance, the numbers of newly confirmed and asymptomatic cases only show a negative correlation with the tourism demand growth rate in the South and Southwest regions of China. The relationship between the number of COVID-19 recovered patients and the tourism demand growth rate differs between the southern and northern regions, showing a weak positive correlation in the Southwest region and a weak negative correlation in the North China region. Notably, a significant moderate negative correlation exists between public medical resources of local source cities and the tourism demand growth rate. In the post-pandemic era, factors related to tourism demand growth rate are constantly changing, and the relationship between influential factors and tourism demand growth rate shows spatial non-stationarity across different regions.

Significant explanatory variables in the East China region include per capita disposable income, average wages, consumer price index, savings deposits, the number of people covered by basic medical insurance, and highway passenger traffic. In South China, the variables include the resident population, per capita GDP, per capita disposable income, per capita consumer spending, savings deposits, PM2.5 concentration, AQI index, newly confirmed cases, newly confirmed asymptomatic cases, the number of hospitals, hospital bed count, basic medical insurance coverage, mobile phone user count, broadband access user count, private car ownership, and civil aviation passenger traffic. In Southwest, the variables include per capita GDP, infection count, newly confirmed cases, newly confirmed asymptomatic cases, recoveries, private car ownership, highway passenger traffic, and spatial distance. In North China, significant variables include savings deposits, industrial solid waste utilization rate, recoveries, mobile phone user count, broadband access user count, and private car ownership. In Central China, the variables include per capita GDP, savings deposits, AQI index, hospital bed count, basic medical insurance coverage, mobile phone user count, broadband access user count, private car ownership, and spatial distance. In Northeast, the significant variables are average wages and savings deposits. In Northwest, no significant explanatory variables have statistically meaningful correlation with tourism demand growth rate.

### Stepwise regressions

Diverging from studies that directly engage in regression analysis with correlation tests^6^, this chapter adopts a stepwise regression modeling approach. It sequentially incorporates significant explanatory variables from Table 3 (highlighted in bold) for each region into the model to explore influential factors under optimal fitting circumstances. However, the results of stepwise regression analysis reveal that in the Northeast and Northwest regions (Table 4), due to a limited number of significant explanatory variables and a lack of significant linear relationships between these variables and the dependent variable, no effective models are obtained. In other regions, there is a noticeable disparity between influential factors and model fit.

**Table 3:** The correlation of scaled proxy variables with TDGR in regions.

Proxy variables	EC	SC	SWC	NC	CC	NEC	NWC
P1	− 0.188	− 0.451**	0.206	− 0.195	0.002	− 0.27	0.12
E1	− 0.164	− 0.361*	0.318*	− 0.285	− 0.470**	− 0.21	− 0.093
E2	− 0.248*	− 0.402*	0.092	− 0.212	0.064	− 0.067	0.208
E3	− 0.066	− 0.393*	0.12	− 0.158	− 0.043	− 0.167	0.109
E4	− 0.314**	− 0.251	− 0.046	− 0.242	0.133	− 0.350*	− 0.029
E5	− 0.225*	0.031	0.002	− 0.074	− 0.278	− 0.13	0.083
E6	− 0.247*	− 0.568**	0.103	− 0.405*	− 0.362*	− 0.390*	− 0.059
EC1	0.014	− 0.455**	− 0.236	− 0.323	− 0.259	− 0.093	0.051
EC2	0.019	− 0.463**	− 0.231	− 0.299	− 0.353*	− 0.101	0.038
EC3	− 0.033	0.241	0.235	− 0.342*	− 0.115	− 0.029	− 0.001
EC4	–	0.243	0.255	− 0.039	0.021	− 0.161	0.311
C1	− 0.085	− 0.231	− 0.377*	− 0.326	− 0.022	0.041	0.032
C2	− 0.046	− 0.342*	− 0.391**	0.147	− 0.031	− 0.172	0.095
C3	0.113	− 0.336*	− 0.375*	− 0.087	− 0.226	− 0.108	− 0.073
C4	0.038	− 0.056	0.155	− 0.284	− 0.055	0	− 0.246
C5	− 0.016	− 0.114	0.334*	− 0.401*	− 0.049	0.067	0.102
H1	− 0.148	− 0.400*	0.211	− 0.185	− 0.284	− 0.141	0.024
H2	− 0.173	− 0.344*	0.227	− 0.236	− 0.363*	− 0.212	− 0.032
H3	− 0.230*	− 0.501**	0.175	− 0.266	− 0.444**	− 0.194	0.064
IA1	− 0.171	− 0.499**	0.25	− 0.357*	− 0.462**	− 0.216	− 0.071
IA2	− 0.183	− 0.453**	0.217	− 0.333*	− 0.532**	− 0.118	− 0.122
TA1	− 0.122	− 0.482**	0.416**	− 0.341*	− 0.607**	− 0.312	− 0.118
**TA2**	− 0.293**	− 0.139	0.349*	− 0.216	− 0.116	− 0.181	− 0.068
TA3	− 0.214	− 0.439**	0.126	− 0.064	− 0.107	0.106	− 0.145
TA4	− 0.17	− 0.143	0.607**	− 0.119	0.345*	0.235	0.132
Sig var	6	16	8	6	9	2	0

* p < 0.05, ** p < 0.01; dependent variable: TDGR; sig var is the number of significant proxy variables in the region.

**Table 4:** The results of stepwise regressions in regions.

Region	EC	SC	SWC	NC	CC	NEC	NWC
Constant	54.056** (7.303)	67.181** (− 7.428)	− 90.459** (− 3.672)	86.833** (− 4.657)	60.366** (− 7.615)	–	–
P1	–	–	–	–	–	–	–
E1	–	–	− 204.613** (− 3.077)	–	− 64.224** (− 3.325)	–	–
E4	− 72.687* (− 2.547)	–	–	–	–	–	–
EC2	–	− 52.889** (− 2.836)	–	–	–	–	–
EC3	–	–	–	− 80.653* (− 2.655)	–	–	–
TA4	–	–	347.222** (5.370)	–	–	–	–
Sample	n = 77	n = 39	n = 43	n = 36	n = 43	n = 35	n = 35
*R*^2^	0.080	0.179	0.519	0.172	0.212	–	–
Adjusted *R*^2^	0.067	0.156	0.482	0.147	0.193	–	–

Dependent variable: TDGR. Independent variables that have no significant linear relationship with the dependent variable in all regions are not retained in the table. *p < 0.05, **p < 0.01, t-values in parentheses.

In East China, average wages are the sole factor affecting the growth rate of tourism demand, albeit with weak explanatory power (R^2^ = 0.08). In South China, the AQI index exerts a negative impact on the tourism demand growth rate, contributing to 17.9% of the variance explanation. In North China, the growth rate of tourism demand is negatively affected by the industrial solid waste utilization rate, with an explanatory power of 17.8%. In Central China, per capita GDP is a significant factor negatively impacting the tourism demand growth rate. In the Southwest region, the tourism demand growth rate is influenced by multiple factors, where spatial distance has a positive impact, and per capita GDP has a significant negative impact. Combined, they account for 42% of the explanatory power concerning the growth rate of tourism demand.

In summation, the growth rate of tourism demand in source markets chiefly hinges on local economic, environmental, and transportation factors. Although there is a significant weak correlation between the local pandemic situation and the tourism demand growth rate in certain areas, it fails to exert a statistically significant impact on the local tourism demand towards the Duku Road.

The efficacy of the models has been corroborated by the diagnostic items and parameters delineated in Table 5. The results indicate that the models passed the F-test (p < 0.05), implying that the independent variables in each model significantly affect the dependent variable TDGR, denoting the models are meaningful. With a limited number of dependent variables in the models, the VIF (Variance Inflation Factor) values are well below 5, suggesting that there is no multicollinearity issue, and the models are well-constructed.

**Table 5:** The results of the model diagnostics.

Diagnostic items	JHT	MT	AT	RT	Results
Diagnostic parameters	F (P < 0.05)	VIF (< 5)	D-W value (1.7, 2.3)	Remodeling	
Model (E.C.)	F (1,75) = 6.485 p = 0.013	E4 = 1	2.21	Consistency	Pass
Model (S.C.)	F (1,37) = 8.042 p = 0.007	EC2 = 1	1.508	Consistency	No
Model (SWC)	F (2,40) = 14.478 p = 0.000	E1 = 1.634 TA4 = 1.634	2.010	Consistency	Pass
Model (N.C.)	F (1,34) = 7.051 p = 0.012	EC3 = 1	1.953	Consistency	Pass
Model (CC)	F (1,41) = 11.057 p = 0.002	E1 = 1	1.721	Consistency	Pass
Model (NEC)	–	–	–	–	No
Model (NWC)	–	–	–	–	No

*JHT* joint hypotheses tests, *M.T.* multicollinearity tests, *AT* autocorrelation test, *RT* robustness tests, *Remodeling* remodeling with the alternative dependent variable, *VIF* variance inflation factor, *D-W Value* white test results.

In the autocorrelation test of the models, the D-W (Durbin-Watson) value of the model for the South China region deviates significantly from 2 (1.508 < 1.7), indicating that the significance testing and goodness of fit for this model would be unreliable; hence, this model failed the validation, while the other models are deemed credible. Moreover, this section re-models using TDR from Sect. 7.2.2 as an alternative dependent variable following the framework procedure. The results of the new model are consistent with the conclusions previously obtained, eventually leading to linear regression models for the tourism demand growth rate in four regions.

### Factors affecting TDGR

**Figure 8:**
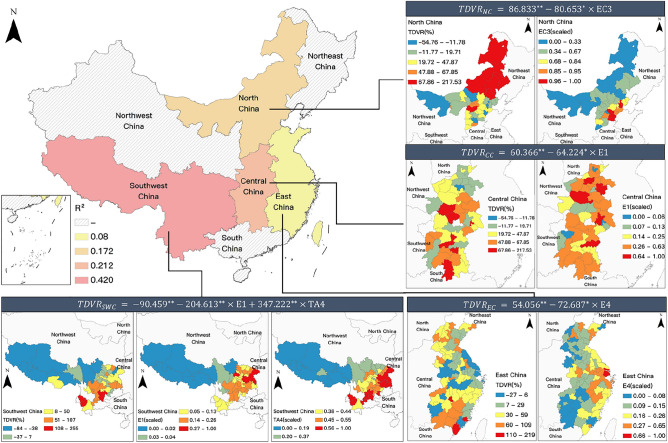
The influential factors models of response heterogeneity of tourist origin markets to Duku Road.

The response to the changes in tourism demand for the Duku Road during the pandemic period is manifested through the tourism demand growth rate of the source cities. The results of the regional model construction discussed earlier (see section "Stepwise regressions") indicate that the factors affecting the growth of tourism demand for the Duku Road vary significantly across different regions. As shown in Fig. 8, on a national scale, the models for the North China, Central China, East China, and Southwest regions have all passed the significance test and robustness test. Each model corresponds to a subplot, with the title of the subplot being the model formula for the respective region, and the content of the subplot displaying the spatial distribution of the tourism demand growth rate and explanatory variables. All indicators are divided into five intervals using the Jenks Natural Breaks classification method, with colors from blue to red representing intervals from low to high.

Upon comparing the similarities and differences across regional models, it is found that during the pandemic period in Xinjiang, the primary factors influencing the response differences to destination lockdowns in the domestic source markets for the Duku Road include Per Capita GDP, average wages, industrial solid waste utilization rate, and spatial distance. Economic factors have a counteractive effect on the tourism demand growth of the source cities, an impact widely present in East China, Central China, and Southwest regions, exhibiting robust spatial consistency. Notably, environmental factors indicate that the growth of tourism demand is constrained by the industrial solid waste utilization rate, a significant relationship found only in North China. This section observes that in industrially prosperous areas within this region (Hebei-Tianjin), the growth of tourism demand is limited, or even decreased, whereas in remote areas with lower industrial levels (Inner Mongolia), the tourism demand is not high, but generally has a high rate of growth. The significant positive impact produced by the distance factor in the Southwest region can be attributed to the distinct differences between the Qinghai-Tibet Plateau and the Sichuan-Chongqing-Yunnan-Guizhou Plateau; the former has low levels of tourism demand and growth rate, while the latter has relatively higher tourism demand and growth rate, thereby leading to a higher tourism demand growth response in source cities farther from the Duku Road.

### Factors influencing tourism demand towards Duku road

Understanding the factors influencing the tourism demand from source cities to Duku Road is crucial for grasping the heterogeneity in tourism demand growth rates. This section employs a loop iteration approach to individually examine the linear fit between twenty-five explanatory variables of source cities within seven administrative regions, and the tourism demand scale of these cities towards Duku Road, to investigate the factors affecting the tourism demand scale of source cities to Duku Road. From a total of 175 linear models, eight models were identified as statistically significant and demonstrating a superior degree of fit ultimately. These models, with a significance level exceeding 0.01 and an R^2^ value greater than 0.8, effectively explain the scale of tourism demand across more than four administrative regions.

As illustrated in Table 6, different variables significantly positively impact the demand for tourism to the Duku Road across various regions. The rise in the number of broadband and mobile phone users suggests more individuals have mobile communication capabilities, enhancing the accessibility to information regarding Duku Road. This improved digital outreach fosters the online visibility and awareness of the destination, consequently boosting the local residents’ desire to travel there. Savings deposits reflect the economic capacity of individuals or families in the source cities, laying the foundation for affording travel to Duku Road. A higher per capita Gross Domestic Product indicates a relatively higher income level and consumer spending capacity, enabling more discretionary income for long-distance travel expenditures. The economic stature of the source cities implies a higher preference for tourism and a more open cultural backdrop, collectively fostering an increased interest and demand for traveling to Duku Road. An augmentation in the number of individuals covered by basic medical insurance and the quantity of hospital beds signifies enhanced medical security for more residents. Better medical services and emergency response capabilities help mitigate the impact of local epidemics on residents’ travel activities. Simultaneously, it alleviates tourists’ concerns about the travel risks and uncertainties associated with the epidemic situation in Xinjiang, bolstering their confidence in traveling to Duku Road. Higher air passenger traffic denotes more flight connections between the residents and the destination, supporting the accessibility for inland tourists to Duku Road. Elevated private car ownership in source cities inclines residents towards choosing self-driving tours to Duku Road, offering greater flexibility, freedom, comfort, and convenience. This mode of travel satisfies tourists’ desire to explore the scenic routes of Duku Road and indulge in personalized experiences.

**Table 6:** The results of the models in regions.

Determinant	Variable	Region
Internet access (R^2^ = 0.869)	Number of mobile phone subscribers + (***)	NEC, EC, CC, NC, SC, NWC, SW
	Number of broadband access subscribers + (***)	NEC, CC, NWC, SW
Economy (R^2^ = 0.918)	Savings DEPOSITS + (***)	NEC, EC, CC, NC, SC, NWC, SW
	GDP per capita + (***)	NEC, SC, NWC, SW
Healthcare (R^2^ = 0.897)	Number of basic medical insurance Participants + (***)	EC, CC, NC, NWC, SW
	Number of hospital beds + (***)	NEC, EC, SC, NWC, SW
Traffic activation (R^2^ = 0.911)	Air passenger traffic + (***)	EC, CC, NC, NWC
	Private vehicle + (***)	NEC, CC, NWC, SW

^+^Signifies that the variable has a positive effect on the tourism demand from source cities to Duku Road, ***p < 0.01.

**Figure 9:**
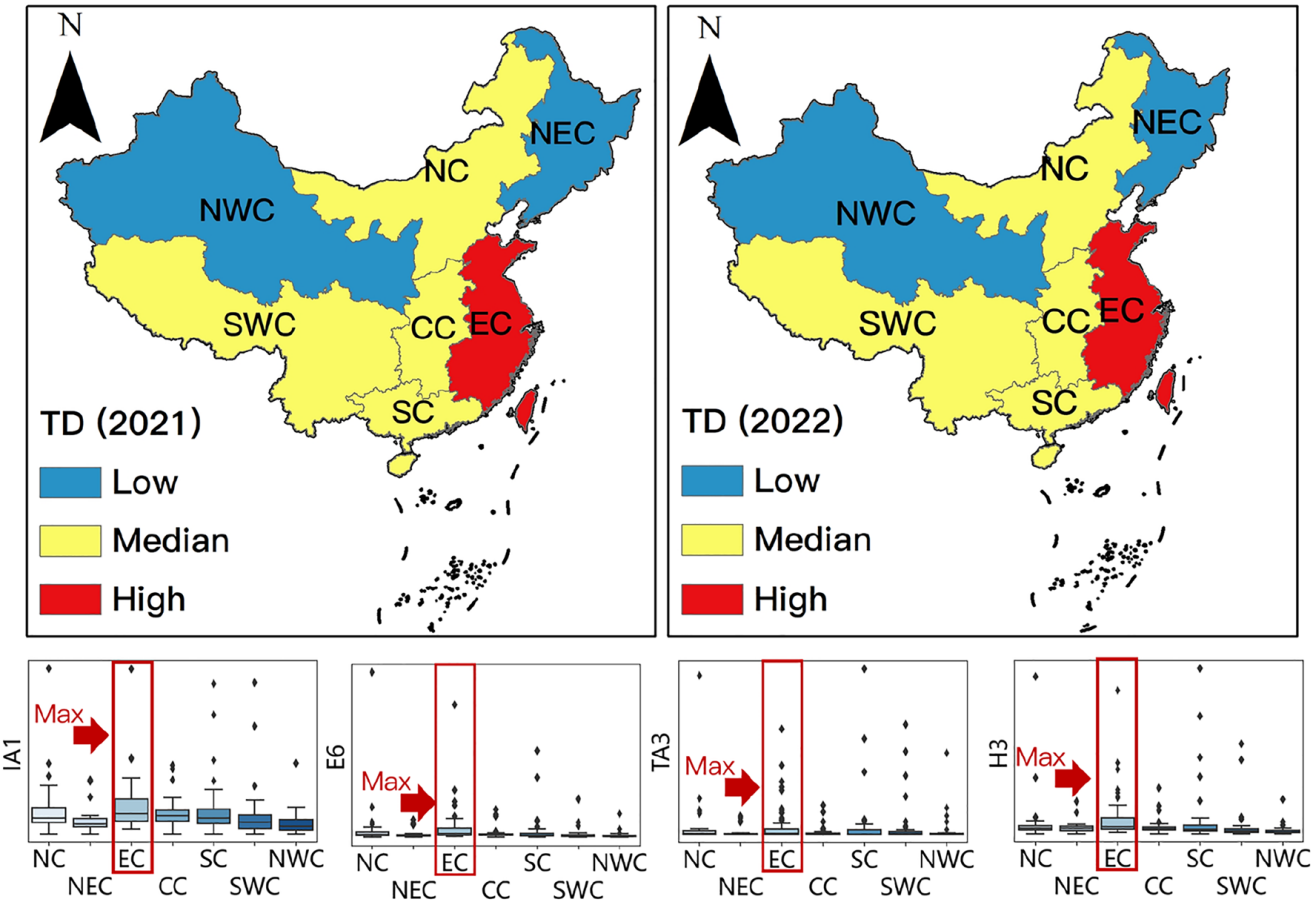
Tourism origins market maps corresponding with boxplots of variables in regions.

In summary, determinants such as the extent of internet coverage, economic conditions, medical security, and transportation facilities in the source markets influence the residents’ demand for tourism to the Duku Road in various aspects. The extent of internet coverage lays the foundation for environmental awareness of the Duku Road among residents of the source cities; whereas economic conditions are essential for residents to travel to the Duku Road. Amid an ongoing pandemic, medical security in the source cities can mitigate the adverse effects of the local epidemic on residents’ travel behaviors; transportation conditions provide diversified accessibility for the residents of the source cities. In the source markets of the Duku Road, areas with a large scale of mobile phone users, high residents’ savings, comprehensive medical insurance coverage, and developed passenger aviation contribute the most to the tourism demand to the Duku Road. This conclusion aligns with the spatial pattern of tourism demand discussed in section "Spatial and temporal changes in origin markets demand". As illustrated in Fig. 9, box plots of the number of mobile phone users (IA1), savings deposits (E6), the number of individuals covered by basic medical insurance (H3), and the volume of civil aviation passengers (TA3) demonstrate that the Eastern China region ranks highest in these four indicators nationwide, and concurrently, it has the highest demand for tourism to the Duku Road. Furthermore, summarizing the determinants in Table 6 and arranging them in descending order based on the average R^2^ reveals that the economic conditions > transportation accessibility > medical conditions > extent of internet coverage, indicating that economic conditions are the most crucial determinant affecting the scale and spatial pattern of tourism demand in the source markets.

## Discussion

### The competitive resilience of core tourist attractions

The study unveils a nuanced understanding of tourism demand dynamics, particularly illuminating the interplay between spatial structures of source markets and economic factors amidst unprecedented health crises such as COVID-19. Even during the crisis, the potential demand from tourist origin markets for a leading tourist attraction continues to be remarkably high and will have considerable potential to counteract the crisis in the post-pandemic era^11^. By delving into the spatial patterns and demand determinants for Duku Road in Xinjiang during the epidemic silent management period, the study extrapolates the driving forces behind the resilience and demand sustenance of core tourism attractions.

Firstly, the notable positive spatial autocorrelation within Duku Road’s source markets elucidates a significant conceptual insight: geographically proximate source cities harbor similar tourism demands. The absence of a Matthew Effect, and the ensuing market dispersion and diversity, underscores Duku Road’s ability to allure visitors from a broad geographic spectrum, thereby diluting over-reliance on singular markets. This diversification not only embodies adaptive capacity to navigate tourism adversities but also buffers the blow from crises by enabling a continuum of demand from unaffected markets. The relative unscathed potential tourism demand during the epidemic crisis manifests the merits of such market diversification.

Secondly, the decisive role of economic factors extends beyond merely shaping the tourism demand from tourist markets towards Duku Road; it critically impacts the spatial configuration of its source markets^58^. The stratified heterogeneity in crisis response, with economically robust core source cities demonstrating stability versus the sensitivity of secondary source cities with moderate economic levels, unveils an inherent inertia in the spatial structure steered by economic dynamics. This is a salient contribution to understanding the spatial-economic nexus in tourism demand studies.

Lastly, the regional variance captured through sectional models divulges that core source cities with high economic indicators face a saturation effect in tourism demand scale for Duku Road, contrasting with potential source cities that possess a larger scope for escalating tourism demand growth rates. It also identifies regional differences in the factors influencing tourism demand variations^19,59,60^. This saturation effect, negatively impacting tourism demand growth rates, accentuates the regional disparities in factors influencing tourism demand variations. The knowledge will help to provide insights into the influential factors driving tourism demand variations in distinct regions^59^.

In synthesis, the resilience of core tourism attractions within a region is constructed on a quadrate foundation: the uniqueness of tourism experience, market dispersion, heterogeneity in crisis response, and spatial structural stability induced by economic factors in source cities. Post-crisis, core attractions sustain a substantial scale of potential tourism demand, with economic factors significantly steering the tourism demand scale, leading to a spatial structural inertia towards core cities. The marked discrepancies in response patterns among source cities, tied to the saturation effects on local economic factors, spotlight the imperative of diversifying source market risks to accrue potential tourism demand, thereby accelerating recovery and sustaining competitive edge post-crisis.

These insights are seminal for policymakers and industry stakeholders to craft efficacious strategies for mitigating crises impacts, fostering tourism recovery, and bolstering resilience. The discourse propels a deeper comprehension of the factors instigating regional variances in tourism demand, furnishing a robust theoretical scaffold for future empirical explorations in the realm of tourism demand studies amidst crises. This narrative, therefore, extends a substantial theoretical contribution towards understanding the ramifications of COVID-19 on tourism demand, while amplifying the criticality of domestic demand in substituting the downturn in international tourism, thereby laying a robust groundwork for successful post-crisis recovery and long-term resilience in regional tourism sectors.

### Limitations and prospects

The limitation in dataset is that the dependent variable does not measure the actual visits, revenues, or employment in tourism in the destination area. Instead, it measures Internet searches for a particular attraction. As Internet interest is indeed correlated with actual trips, the possibility of inferring short-term variations in demand on the search alone is limited. Regarding our emphasis on core tourist attractions within a regional destination has inadvertently disregarded the vulnerabilities of a broader array of regional attractions. Forthcoming studies will encompass an evaluation of the comprehensive repercussions that tourism crises exert on the destination system, alongside an analysis of the source market’s reactions. Additionally, studies will classify tourist attractions by their level or type and will subsequently discuss the risk resilience and the mitigating strategies employed for each group of tourist attractions. An integrated and differentiated methodology such as this promises to yield a more systematic understanding, benefiting the strategic planning and operational decisions of tourism management entities.

In contemplating future research directions, several potential areas warrant further investigation. Initially, a novel comprehensive indicator should be generated for monitoring tourism demand by integrating search data, user-generated content on social media platforms, and statistical data. This integration would avoid the original error in sample coverage of the source market due to internet access disparities. Furthermore, expanding the research framework to encompass additional destinations and broader dimensional factors, and employing mixed-effect models to study the overall impact of these factors on tourism demand, would yield a more holistic understanding of tourism demand dynamics during crisis situations. Additionally, examining the economic downturn in the post-epidemic era, with a particular focus on the influence of decreased travel intentions following the lifting of travel restrictions and the subsequent general reopening, could offer crucial insights into the resilience of various destinations and origin markets. Lastly, an in-depth analysis of the long-term consequences of crisis events on tourism demand, as well as the recovery trajectories of destination and origin markets, would be instrumental in providing essential guidance for policymakers and industry planners aiming to mitigate the adverse effects of crises on the tourism sector.

## Conclusions

This inquiry delved into the dynamics of potential tourism demand from China's domestic origin markets towards Duku Road in Xinjiang during the epidemic closure in August 2022. The exploration unfolded heterogeneous response patterns of tourism demand in the face of unexpected epidemic crises, particularly accentuating the post-epidemic era. The study homed in on the impact of demographic, economic, environmental, epidemic, medical, digital, and transportation facets on local tourism demand fluctuations within the origin markets, encompassing 308 cities. Utilizing spatial statistical and stepwise regression analyses, the investigation spotlighted spatial disparities in tourism demand variation rates across seven major regions.

A salient revelation is the altered response patterns of origin markets to epidemic crises at destinations, transcending the initial reactions during the 2019 onset of COVID-19. There is a notable uptick in tourism demand gravitating towards primary origins. While local epidemics and medical care in origins correlate with tourism demand variations, they do not forge a meaningful relationship. Economic determinants emerge as dominant negative influencers, with tangible regional disparities in factors affecting local tourism demand rates. Economically affluent regions surface as the core tourist origin markets, exhibiting resilience in tourism demand amidst destination tourism crises. Concurrently, core tourist-origin areas with high economic indicators appear to reach a saturation point, curtailing the growth rate of tourism demand. Conversely, potential tourist origin markets highlight significant variability with a pronounced growth potential, revealing a correlation with distance and an inverse relationship with industrial solid waste utilization rate.

Central to the findings above is the competitive resilience of core tourism attractions like Duku Road, demonstrated by its ability to maintain a stable tourism demand even amidst adversities such as the epidemic crisis. Firstly, the study demonstrates that core tourist attractions within destination systems can accumulate potential tourism demand during crises through their intrinsic appeal and diversified market structure. This accumulated demand provides substantial momentum for recovery, highlighting the importance of maintaining and enhancing the attractiveness of core tourism assets to sustain potential tourism demand. Secondly, core tourist attractions serve as both growth poles within destination systems and recovery nodes for tourism revival. The spatial differentiation and clustering of tourism demand driven predominantly by economic factors within origin markets contribute significantly to the risk resilience of core attractions. These factors suggest that economically robust regions can act as stable sources of tourism demand, reinforcing the importance of understanding and leveraging economic conditions in origin markets to enhance the resilience and competitive edge of core attractions.

The insights are pivotal for stakeholders aiming to design efficacious strategies to navigate through crises, promoting tourism recovery and resilience, thereby maintaining the competitive edge of core tourist attractions, and regional tourism sustainability by strategies as followed: (1) Destination management organizations should focus on diversifying their market structures to include a mix of economically strong and emerging markets to buffer against localized crises. (2) Continuous monitoring of economic indicators in origin markets can provide early warnings and strategic insights for tourism demand management, allowing for more resilient destination planning. (3) Policymakers should prioritize strategies that enhance the core attractiveness of key tourism assets while simultaneously fostering diversified and resilient origin markets to safeguard against potential crises.

## Data Availability

The raw datasets utilized in this study can be accessed through the Baidu search engine at https://index.baidu.com and the National Bureau of Statistics of China at http://www.stats.gov.cn/. The processed datasets that were used and analyzed during this research are available from the corresponding author upon reasonable request.
